# Elucidating the limiting factors for regeneration and successful establishment of the thermophilic tree *Ziziphus spina-christi* under a changing climate

**DOI:** 10.1038/s41598-020-71276-4

**Published:** 2020-08-31

**Authors:** Yotam Zait, Irit Konsens, Amnon Schwartz

**Affiliations:** The Robert H. Smith Institute of Plant Sciences and Genetics in Agriculture, Faculty of Agriculture, Food, and Environment, The Hebrew University of Jerusalem, Rehovot, Israel

**Keywords:** Plant ecology, Plant physiology, Plant stress responses

## Abstract

Due to climate change, winter temperatures are predicted to increase worldwide. For thermophilic trees, highly sensitive to low temperatures, an increase in winter temperatures may be beneficial for survival and regeneration. *Ziziphus spina-christi* is a thermophilic tree that has recently become more abundant and widespread in the eastern Mediterranean, presumably due to a gradual increase in winter temperatures. We aim to define the temperature limitations for seed germination and the growth and survival of young seedlings to broaden our understanding of the future geographical distribution of this species. We studied effects of temperature on germination, growth, and photosynthesis in a controlled environment with four different day/night temperature regimes (34/28 °C, 28/22 °C, 22/16 °C and 16/10 °C). Effects of endocarp on germination and seed germination in the field were also studied. Results showed that germination has a lower thermal optimum (34–22 °C, 63.5–67.5% germination) than growth and photosynthesis (34–28 °C). Moderate cold stress (22/16 °C), did not affect germination capacity, but strongly reduced seedling growth (71%) and photosynthetic capacity (44.6%). Under severe cold stress (16/10 °C), germination still occurs (22%), but seedlings cannot perform growth and photosynthesis. We conclude that slow seedling growth, not germination, is the main barrier for successful establishment of *Z. spina-christi* under low temperature. Warmer winters could lead to earlier establishment of seedlings and increase their chance of survival the following summer. This may explain the recent increase in the tree’s relative abundance and further highlight the potential spread of this species at higher altitudes and latitudes across the Mediterranean.

## Introduction

Future climate change models predict that many regions of the world will experience shifts in temperature and an increase in the frequency and severity of droughts^1,2^. Over the last century, average temperatures have increased by 1.5–4 °C in the Mediterranean area, with global climate models predicting average warming of 4–6 °C over the next 50–90 years^3^. Additionally, forecasts predict an increase in the frequency and strength of extreme weather events such as drought years, floods, and heat waves over the next 50 years^2^. Climate change may cause species to change their biogeography by either reduce or expand distribution^4^. For thermophilic trees, highly sensitive to low temperatures, an increase in winter temperature may be beneficial for survival and regeneration. For instance, over the past few decades, climate warming has caused thermophilic broadleaved tree species in northern Sweden to expand their geographical distribution^5^. Another group of “thermophilic trees” predicted to be strongly affected in the coming decades by climate change are the African savanna trees^6–8^. African savanna trees evolved a strong capacity of acclimation and adaptation to drought^9^, extreme temperature and radiation^10,11^, and increase of atmospheric CO_2_^12^, but as thermophilic trees they lack mechanisms to cope with low temperatures. Many African native species extended their natural distribution from Sudan and Ethiopia to North Africa and the eastern Mediterranean region^13^. Among the African savanna trees in the eastern Mediterranean, those belonging to the east Sudanese chorotype (such as *Calotropis procera*, *Moringa peregrina*, *Salvadora persica*, *Cordia sinensis*, *Ficus carica*, *Acacia tortilis*, and *A. raddiana*), the *Ziziphus spina-christi* (Rhamnaceae) considered to be the “least cold-sensitive”, since they grow further north in the African-Syrian transform (Great Rift valley)^14^. Additionally, recent observations and studies suggest that the *Z. spina-christi*, has become more abundant in the east Mediterranean, presumably as a result of a gradual increase in winter temperatures^11^. *Z. spina-christi* , is considered resistant to drought, soil salinity, and heat; it survives in desert areas with annual rainfall of only 50–300 mm^11,15–18^. As a thermophilic tree, it cannot survive in places where winter temperatures fall below freezing^13^.

*Ziziphus spina-christi* is cross-pollinated, the cymes developing in the leaf axils of young branches, and the edible fruits, which have a fleshy mesocarp, usually ripen under warm conditions^19^. The tree is propagated through seeds covered with a hard woody endocarp (seed coat) that inhibits water absorption, thereby imposing physical dormancy (also known as exogenous dormancy) that limits germination^20–22^. Under natural conditions, successful germination might be enhanced by the fruit’s passage through an animal’s digestive tract^23^. In certain areas, grazing of the young seedlings limits the proliferation of this species^24^.

Temperature plays an essential role in determining the yearly cycle of seed germination, thereby limiting the *Z. spina-christi*’s geographic distribution. The native tree region (Sudanian savanna) is characterized by a dry tropical climate with relatively high temperatures throughout the year, absence of freezing temperatures, and precipitation mainly during the summer (June–September). In this region, seeds germinate in the summer when soil moisture levels and temperatures are both high. However, the Mediterranean region is characterized by very different climatic conditions, with dry, hot summers (May–October) and cooler, wet winters. Consequently, in the Mediterranean region, *Z. spina-christi* seeds germinate during the winter when soil moisture is high, but temperatures are relatively low. This forces the seedling to cope with occasional chill events, which can be lethal. The relatively slow growth of the seedlings during the first year (mainly of the shoot) may be another limiting factor on the successful establishment of the saplings. For these reasons, *Z. spina-christi* may be among those trees that might benefit from future warming. Yet, there is a lack of information regarding the effect of temperature on the germination of *Z. spina-christi* seeds and the growth of the young *Z. spina-christi* seedlings. We hypothesize that warmer winter temperatures may have a beneficial influence on germination and the survival of young seedlings during the following summer.

An additional limiting factor for *Z. spina-christi’s* successful establishment is its incapacity to photosynthesize and respire at low temperatures, therefore limiting the production of sugars necessary for growth and other metabolic maintenance processes. We previously showed that even at low temperatures, the tree keeps relatively high stomatal conductance (*g*_*s*_) and, therefore, diffusion limitation of CO_2_ trough stomata playing a minor role in photosynthesis^16^. The diffusional conductance of CO_2_ through the leaf internal mesophyll cells (i.e., mesophyll conductance, *g*_*m*_) was found to be the key limiting factor to photosynthesis under low temperature. The dependence of photosynthesis on temperature has been extensively studied in many tree species, but there is still missing knowledge in thermophilic trees in general, and *Z. spina-christi* in particular.

The goal of the present study is to define the temperature limitations for seed germination and the growth and survival of young *Z. spina-christi* seedlings and to broaden our understanding of the nature of the future geographical distribution of this species. We also aim to understand the reason(s) for the greater abundance and the broader distribution of this species over the last few decades.

## Materials and methods

### The effect of temperature on germination

Dry *Z. spina christi* fruits collected in a single location were obtained from a public nursery (JNF; Beit Nehemiah, Israel). The pulp of the fruit was removed to reveal the woody endocarp that encloses a single seed. The germination experiment was conducted in a greenhouse (phytotron) that included four rooms kept at a different day/night temperatures: 34/28 °C, 28/22 °C, 22/16 °C and 16 °C/10 °C. Seeds were sown in 0.5-L pots filled with a 1:1 mixture of vermiculite (No. 2) and tuff gravel. Each treatment consisted of 10 pots, with 20 seeds in each pot. The seeds were checked carefully to verify that they were undamaged. Seeds were sown at a depth of 2 cm above a piece of gauze to verify that the depth of the seeds remained unchanged. Shade nets (providing 75% shade) were positioned above the pots to avoid any effects of high radiation on germination. After germination, the shade nets were removed, and seedling growth was monitored. Stem elongation and the rate of leaf appearance were monitored on the main stem every 2–5 days by tagging the new first defined leaf close to the apical meristem on each observation day. The seedlings were irrigated twice a day to guarantee an unlimited water supply. The irrigation solution contained the following concentrations of specific nutrients: 2.0 ± 0.1 mM Ca, 1.2 ± 0.2 mM Mg, 0.27 ± 0.04 mM NH_4_, 4.6 ± 0.1 mM NO_3_, 0.30 ± 0.02 mM P and 2.3 ± 0. 2 mM K.

### The effect of the woody endocarp (seed coat) on germination

This experiment was conducted in the controlled-environment greenhouse with day/night temperatures of 28/22 °C. This experiment included five treatments: seeds with a fully intact endocarp (control), seeds whose endocarp was removed, seeds whose endocarp was cracked (with a soft blow from a hammer), seeds that had been subjected to 60 min of scarification with 97% sulfuric acid, and seeds that had been subjected to 120 min of acid scarification. Each treatment consisted of 12 pots, and ten seeds were sown in each pot. This experiment was conducted in the same way as the temperature experiment described above.

### The effect of temperature on the growth of young saplings

Saplings were grown in the greenhouse, as described above, in 10-L containers filled with highly porous organic planting soil. To allow for gradual acclimation to the different growth conditions, plants were grown for 1 month at day/night temperatures of 28/22 °C and then transferred to the different temperature treatments: 34/28 °C, 22 /16 °C and 16/10 °C. Stem elongation and rate of leaf appearance were measured every 2–5 days. On each observation day, the first defined leaf close to the apical meristem was tagged, the new leaves were counted, and the length of the new internodes was measured. The total number of new leaves on the main stem and the lateral branches was counted from the beginning of the experiment. Specific leaf area and total leaf area were measured at the end of the experiments, following defoliation by hand, using a leaf-area meter (model LI-3100, LI-COR, Lincoln, NE, USA). At the end of the experiment, the different parts of the plant (root, branches and leaves) were separated, dried in an oven at 70 °C and then weighed. Stem cross section were sampled from every treatment 10 cm from the shoot apical meristem.

### Gas exchange and chlorophyll *a* fluorescence measurement

Gas exchange measurements were conducted using the LI-6400 photosynthesis measurement system (LI-COR Inc., Lincoln, NE, USA), equipped with a 2 cm^2^ fluorescence leaf chamber. Measurements were conducted at a saturating light intensity 1,500 μmol photons m^−2^ s^−1^ for, with air flow of 500 μmol air s^−1^. The block temperature was set according to the different room temperature treatments, and the relative humidity was set to 50% using the LI-6400 desiccant. For all gas exchange we chose the youngest fully expanded leaf (leaf 5–7). After a leaf was clamped in the LI-6400 chamber, we allowed 5–15 min of acclimation to a fixed flux of 400 μmol CO_2_ mol^−1^ air. Then, CO_2_ response curves for gas exchange combined with chlorophyll a fluorescence were measured. The initial CO_2_ concentration was 400 μmol CO_2_ mol^−1^ air, which was then reduced to 300, 200, 150, 100, and 50 ppm. The CO_2_ concentration was then returned to 400 μmol CO_2_ mol^−1^ air followed by an increase to 600, 800, 1,000, 1,200 μmol CO_2_ mol^−1^ air.

Mesophyll conductance (*g*_*m*_), was estimated using the variable J method^25^:

$${g}_{m}=\frac{A}{{C}_{i}-\frac{{\varGamma }^{*}*(J+8*(A+{R}_{d})}{J-4*(A+{R}_{d})}}$$

where *R*_*d*_ is the non-photorespiratory respiration in light and ${\varGamma }^{*}$ is the apparent photo-compensation point estimated according to the “Laisk method”^26^. J is the electron transport rate calibrated from CO_2_-response and light-response curves in the absence of photorespiration (1% O_2_ in the mixed air). In brief: from the response curve measured at 1% O_2_, we performed a linear regression of actual quantum efficiency of photosystem II, ΦPS2^27^, and the quantum yield of CO_2_ fixation (ΦCO_*2*_) to obtain a regression coefficient (k). The theoretical model and experimental observations were limited to the linear region of ΦCO_2_ < 0.05, and ΦPS2 < 0.5. The slope of the linear regression (k) and the y-axis intercept (b) were used to recalculate the (α ∗ β) for the electron-transport rate (J) based on chlorophyll fluorescence measured at 21% O_2_^28^ as:

$$J{{calibrated= 4*(({\varnothing }PS}_{2}-b)/k)*PPFD}_{i}$$

${\alpha }$ is leaf absorptance and β is the proportion of photons absorbed by PSII. The CO_2_ concentration in the chloroplast stroma (*C*_*c*_) was then calculated with the observed *g*_*m*_ value according to Fick’s first law:

$$C_{c} = C_{i} - \frac{{A{\text{n}}}}{{g_{m} }}.$$

A_n_/Ci and A/C_c_ curves were fitted using the “Plantecophys” R Package for analyzing and modeling leaf gas exchange data^29^.

### Photosynthesis short-term acclimation study

To study how the tree response to fast changes in temperature we performed CO_2_ response and chlorophyll fluorescence measurements for plants that grew in the warm room (34/28 °C), and in the cold room (16/10 °C) Then, plants were transferred from the warm room (34/28 °C) to the cold room (16/10 °C), and from the cold room (16/10 °C) to the warm room (34/28 °C), and gas exchange and chlorophyll a fluorescence measurements were taken 1 day and 1 week after the transition.

### Measurements of water and osmotic potentials

The water potential of the leaves—*Ψ*_*w leaf*_ was measured using pressure chamber (ARIMAD 3,000, MRC, Holon, Israel) as described in Zait et al.^16^. In day 45, leaf and root samples were taken from each temperature treatment (n = 5) and immediately transferred into liquid nitrogen. The frozen samples were quickly thawed and transferred into 0.5-ml Eppendorf tubes and centrifuged to extract the tissue sap. The osmotic potential (*Ψ*_*s*_) was determined using a vapor pressure osmometer (Model 5600, ELITech Group, Puteaux, France).

### Field germination study

This study was conducted at the Neot Kedumim Park, a nature reserve located near the town of Modi'in, Israel (31° 56′ 52.3″ N 34° 58′ 23.1″ E). The park covers an area of 25 km^2^ and is characterized by a Mediterranean climate with an average annual precipitation of 520 mm year^−1^ (November–April) and a dry summer. *Z. spina-christi* seeds in their woody endocarps were sown in several plots in January. The experiment included two treatments: seeds with a fully intact endocarp were sown in unshaded areas, 2–10 m from an existing *Z. spina-christi* tree, and in a shaded area underneath the canopy of the *Ziziphus* trees. Each treatment included ten random blocks with 20 seeds per block. All seeds were sown in the soil at a depth of 2 cm and covered with nets to protect them from grazing animals and birds. The germination and development of the seedlings were monitored as were the environmental conditions.

### Data and statistical analysis

Statistical analysis was performed using JMP software (JMP 14 software, SAS Institute Inc., Cary, NC, USA). Significant differences between the seed-germination treatments and the different treatments used in the experiment to evaluate the effect of temperature on sapling growth were determined using the Tukey–Kramer post hoc test. Student's *t*-test (*n* = 10, *P* < 0.05) was used to evaluate differences in the germination rates observed in the different treatments in the field experiment.

## Results

### The effect of temperature on germination and seed emergence

**Figure 1:**
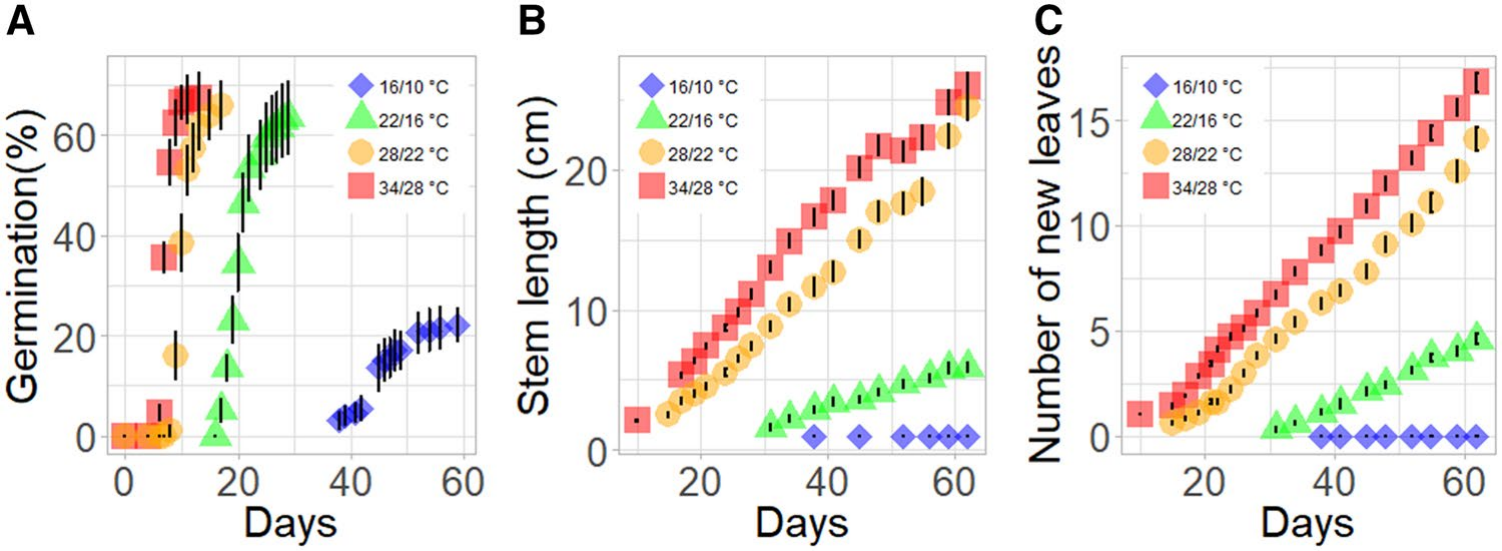
(**A**) The effects of different temperature regimes on the proportion of seeds that germinated, (**B)** seedlings stem length as a function of days from sowing, and (**C**) the number of new leaves as a function of days after sowing.

Germination of *Z. spina-christi* seeds depends on both temperature and physical dormancy. By partially or completely removing the hard endocarp that encloses the seed, we were able to demonstrate that temperature affects dormancy, germination and seedling establishment. The growth temperature had a significant effect on the germination of *Z. spina-christi* seeds (Fig. 1). The germination of these seeds is epigeic; during germination, the hypocotyl elongates first. The date of germination was defined as the day on which the hypocotyl hook first emerged from the soil. The earliest germination, at 6 days after sowing, was observed in seeds exposed to the warmest temperatures (34/28 °C). In the 28/22 °C, 22/10 °C and 16/10 °C treatments, germination started at 8, 17 and 32 days after sowing, respectively.

In the 34/28 °C treatment, maximum germination of 67% of the seeds was reached 7 days after the appearance of the first seedling. In the 28/22 °C treatment, maximum germination (66%) was observed after 9 days. In that treatment, germination continued over a period of 12 days and the maximum germination was 63.5%. In the lowest-temperature treatment (16/10 °C), germination continued for 14 days and only 22% of the seeds germinated. The rate of seed germination in the coldest treatment was significantly lower than that observed in the warmer treatments. After the initial germination, the rates of stem elongation in the 34/28 °C treatment and the 28/22 °C treatment were 0.46 and 0.44 cm day^−1^, respectively (Fig. 1A; Table 1). The rate of seedling growth in the 22/16 °C treatment (0.135 cm day^−1^) was 71% slower than that observed among the seedlings exposed to the warmer treatments. In the coldest treatment (16/10 °C), no stem growth was observed. Like stem elongation, the rate of leaf appearance was also highly dependent on temperature (Fig. 1B; Table 1). The highest leaf-appearance rate was observed in the warmest treatment (34/28 °C; 0.314 new leaves day^−1^). The leaf-emergence rate was 10% lower in the 28/22 °C treatment (0.282 new leaves day^−1^) and 50% lower in the 22/16 °C treatment (0.139 new leaves day^−1^). No new leaves emerged in the coldest (16/10 °C) treatment.

**Table 1:** Seedling stem-elongation rates calculated from the linear slope of the relationship between stem length and the number of days from germination.

Temperature day/night	Stem-elongation rate (cm day^−1^)	*P *value	*R*^2^	Leaf-emergence rate (new leaves day^−1^)	*P* value	*R*^2^
34/28 °C	0.464 a	< 0.0001	0.98	0.314 a	< 0.0001	0.99
28/22 °C	0.446 a	< 0.0001	0.98	0.282 b	< 0.0001	0.99
22/16 °C	0.135 b	< 0.0001	0.99	0.139 c	< 0.0001	0.99
16/10 °C	0c			0d		

Leaf-appearance rate was calculated from the linear slope of the relationship between the number of new leaves and the days from germination, within the different temperature treatments. Different letters denote a statistically significant difference between treatment means identified using the Tukey–Kramer post hoc test.

### The effect of endocarp on germination

**Figure 2:**
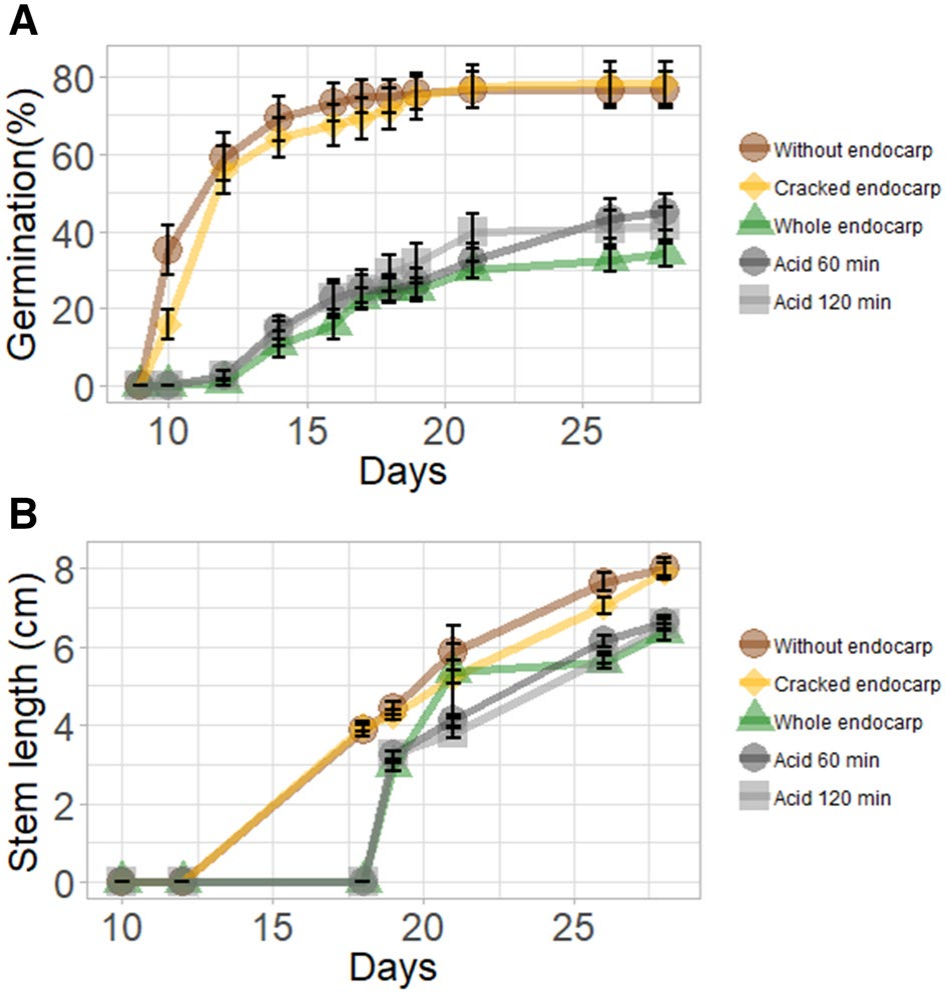
The effects of different acid-scarification treatments, endocarp removal and a cracked endocarp under 28/22 °C day/night temperature regime, on (**A**) the proportion of seeds that germinated and (**B**) stem length as a function of the number of days after sowing.

Seeds with an intact seed coat (control) started to germinate after 12 days from sowing, reaching 34% germination after 26 days. Seeds whose woody seed coats had been removed or cracked with a hammer started to germinate at 10 days after sowing, 2 days earlier than the untreated seeds (Fig. 2A). Seeds whose seed coats had been removed or cracked reached similar maximum germination of 76% at 7 and 9 days after the appearance of the first seedling, respectively.

The acid scarification did not affect the time of germination but did improve the percentage of germination by 8–11%, as compared to the control treatment. The seeds that were immersed in 96% sulfuric acid for 120 min before sowing reached a maximum germination rate of about 40% at 11 days after the appearance of the first seedling. Seeds that were subjected to the 60-min acid treatment achieved maximum germination of 45% at 15 days after germination started. The rate of stem elongation did not differ significantly between the two acid-scarification treatments (Fig. 2B).

### The effect of temperature on the growth of young saplings

**Figure 3:**
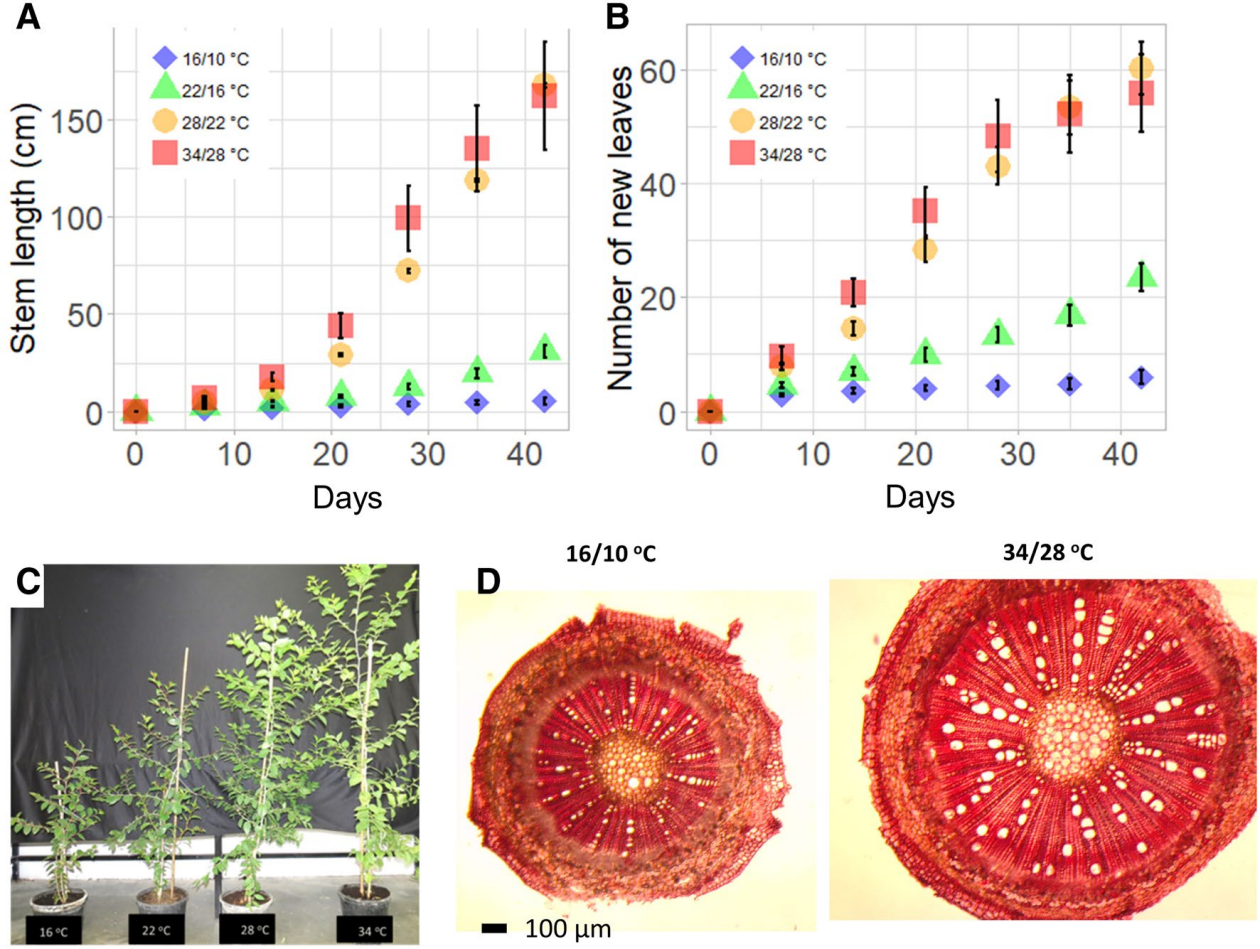
The effect of growth temperature on (**A**) the length of the saplings’ main stems and (**B**) the number of new leaves. (**C**) Image showing saplings after 45 days of exposure to different temperature conditions. (**D**) Image showing stem cross section of young saplings in the cold (16/10 °C) and the warm (34/28 °C) growth temperatures treatment.

Growth of *Z. spina-christi* saplings was monitored in the greenhouse under four different temperature regimes. The measurements began when the saplings were about 6 months old. The plants were grown for about 1 month in the greenhouse at 28/22 °C before they were subjected to different temperature treatments. In the two high-temperature treatments, 34/28 °C and 28/22 °C, the main stem grew at an average rate of was 1.4 cm day^−1^ (Fig. 3). The growth rate was 65% lower (0.5 cm per day^−1^) in the 22/16 °C treatment and 93% lower in the 16/10 °C treatment (0.1 cm day^−1^). In the 34/28 °C and 28/22 °C treatments, new leaves appeared on the main stem at a rate of 4 leaves day^−1^. In the most cooling treatment (16/10 °C), an average of 0.1 new leaves appeared each day; this figure is 97.5% lower what was observed for the high-temperature treatments. Also, a significant decrease in stem diameter (10–16%), vessel diameter (22–26%), vascular area (6–25%), and increased in vessel density (35–76%) were obtained in the cold treatment (16/10 °C) compared to the warmer treatments (Table 2; Fig. 3D).

**Table 2:** The effects of different temperature regimes on the anatomic variables of the xylem.

Temperature day/night	Stem diameter (µm)	Pith diameter (µm)	Vessel diameter (µm)	Vascular area %	Vessel density (1/mm^2^)
34/28 °C	1,716.1 a	694.1 a	32.19 a	38.22 a	1.7 a
28/22 °C	1,836.7 a	886.8 a	30.68 a	30.53 ab	1.37 ab
22/16 °C	1,830.9 a	766.5 a	31.66 a	30.5 ab	1.57 ab
16/10 °C	1,554.9 b	591.7 a	24.1 b	28.84 b	2.3 b

Different letters denote a statistically significant difference between treatment means identified using the Tukey–Kramer post hoc test (*P* < 0.05).

**Figure 4:**
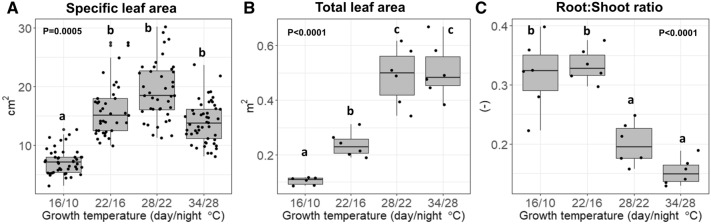
The effect of growth temperature on (**A**) the saplings specific leaf area (SLA), (**B**) total leaf area (TLA), and (**C**) root-to-shoot dry mass ratios. Different letters represent a significant difference between treatments, as determined using the Tukey–Kramer post hoc test.

The specific leaf area of the plants in the lowest-temperature treatment (16/10 °C) was about 50% smaller (7.3 cm^2^) than that observed among the plants exposed to higher temperatures (Fig. 4A). The total leaf area was 0.5 m^2^ among the saplings in the 34/28 °C and 28/22 °C treatments and was about 50% smaller in the 22/16 °C treatment and 80% smaller in the 16/10 °C treatment (Fig. 4B). Lower temperatures caused saplings to invest more in root mass than in shoot mass (Fig. 4C). A root-to-shoot ratio (based on dry mass) of about 30% was observed for the plants exposed to the 22/16 °C and 16/10 °C treatments; that ratio is about 15% higher than the ratio observed among the plants exposed to the 28/22 °C and 34/28 °C treatments.

### The effect of temperature on photosynthesis

**Figure 5:**
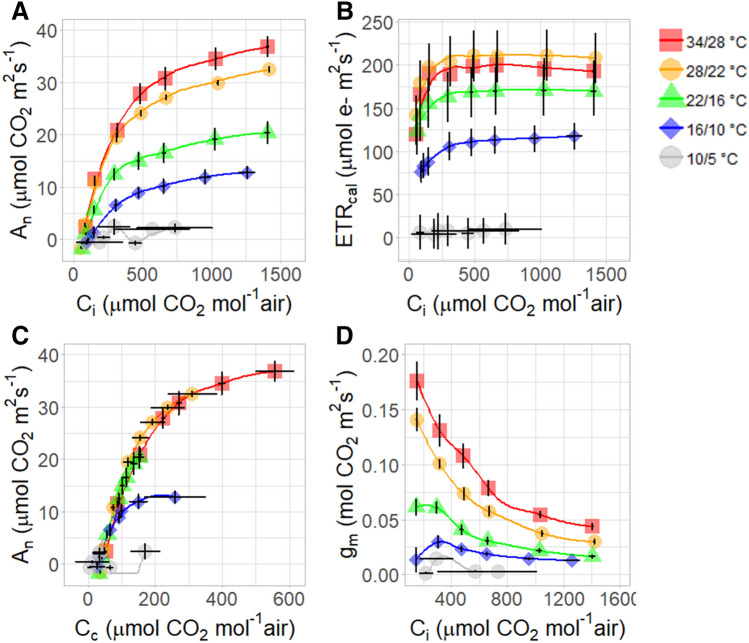
Effect of different growth temperatures on the saplings: photosynthesis rate response to the increase of CO_2_ in the intercellular airspaces (*C*_*i*_) (**A**). Photosynthesis rate (*A*_*n*_) response to the increase of CO_2_ in the chloroplast (*C*_*c*_) (**B**). Mesophyll conductance of CO_2_ (*g*_*m*_) response to the increase of CO_2_ in the intercellular airspaces (*C*_*i*_) (**C**), and the calibrated electron transport rate (ETR_*cal*_) response to the increase of CO_2_ in the intercellular airspaces (*C*_*i*_) (**D**).

Growth temperature has a substantial effect on leaf gas exchange and chlorophyll a fluorescence (Fig. 5). It can be seen from the photosynthesis response to change in the CO_2_ concentration in leaf intercellular airspaces (*C*_*i*_), and CO_2_ concentration in chloroplast stroma (*C*_*c*_) that increasing temperature has a positive effect on photosynthesis performance (Fig. 5A,B). The maximum light-saturated photosynthetic rate (*A*_*max*_) was 36.7 µmol CO_2_ m^−2^ s^−1^ in the 34/28 °C treatment, and decreased to 32.5 µmol CO_2_ m^−2^ s^−1^ (reduction of 11.4%), 20.3 µmol CO_2_ m^−2^ s^−1^ (reduction of 44.6%), 12.7 µmol CO_2_ m^−2^ s^−1^ (reduction of 65.3%) and 2.1 µmol CO_2_ m^−2^ s^−1^ (reduction of 94.2%), for the 28/22 °C, 22/16 °C, 16/10 °C, and 10/5 °C treatments respectively (Fig. 5A,B). Rubisco maximum capacity for carboxylation (*V*_*cmax*_) (derived from the initial slope of the *A*_*n*_/*C*_*i*_ curves and normalized to 25 °C) was highest in the 34/28 °C and the 28/22 °C treatments (89 and 95 µmol CO_2_ m^−2^ s^−1^ respectively), decreased by 23.5% in the 28/22 °C treatment (73 µmol CO_2_ m^−2^ s^−1^), and by 31.5% in the 16/10 °C treatment (65 µmol CO_2_ m^−2^ s^−1^). As can be inferred from the chlorophyll a fluorescence measurements (Fig. 5C), the calibrated electron transport rate (ETR_cal_) increased with the increase of *C*_*i*_ and was highest in plants grown under high temperatures (198 and 210 µmol e- m^−2^ s^−1^ for the 34/28 °C and 28/22 °C treatments respectively), and decreased by 36%, 44% and 100% in the 22/16 °C, 16/10 °C and 10/5 °C treatments respectively. Mesophyll conductance to CO_2_ (*g*_*m*_) was strongly dependent on growth temperature (Fig. 5D), showing relatively high values in the warm treatments (0.175 and 0.15 mol m^−2^ s^−1^ for the 34/28 °C and the 28/22 °C respectively), and decreased by 65%, 82% and 100% in plants growing at the 22/16 °C, 16/10 and 10/5 °C treatments respectively. Interestingly, *g*_*m*_ decreased rapidly with the increase in CO_2_ only in plants that grew under high temperatures (34/28 °C and 28/22 °C), while in plants that grew under low temperatures, *g*_*m*_ showed hyposensitivity to CO_2_.

**Figure 6:**
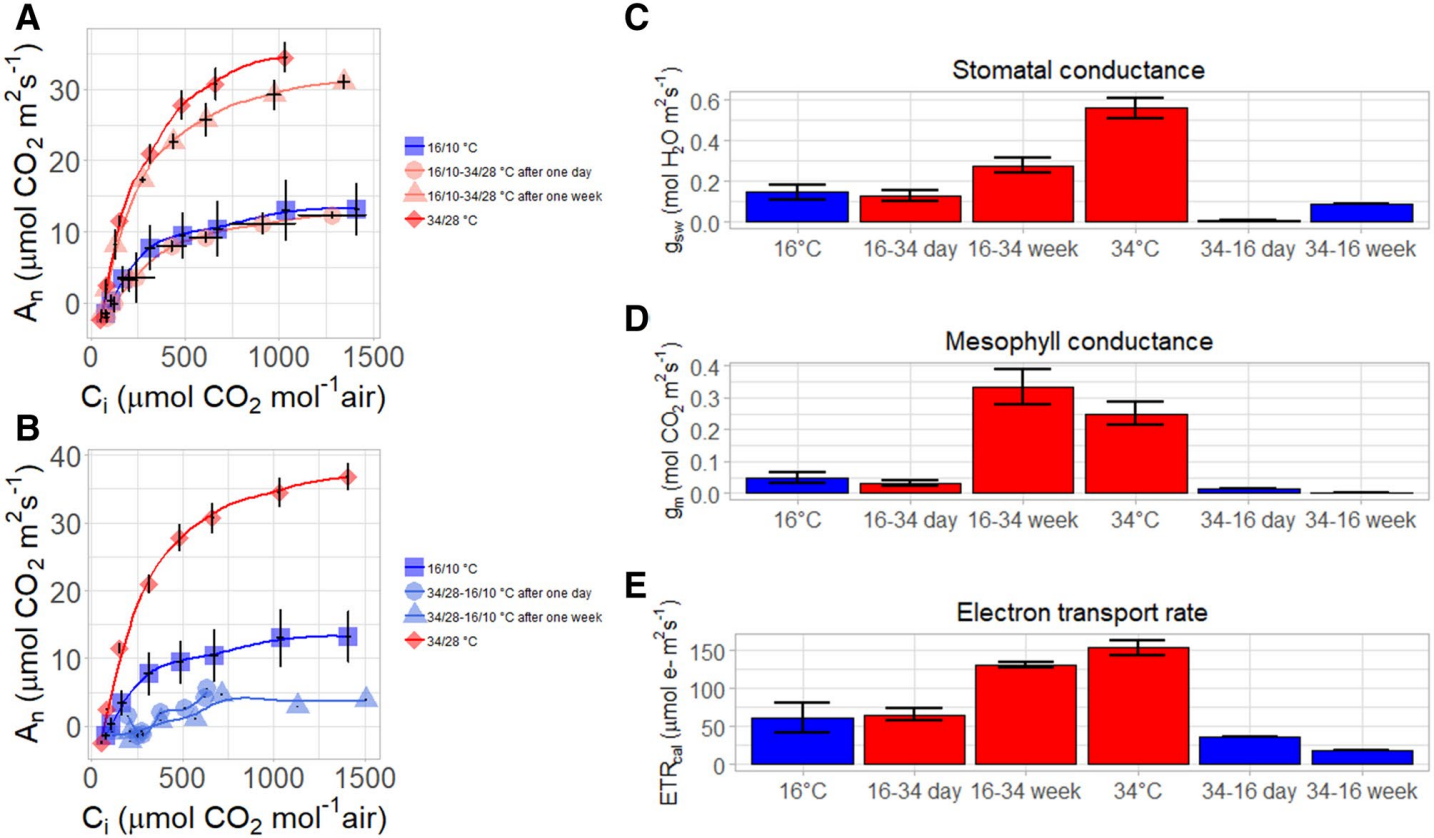
The effect of saplings transition from the cold room (16/10 °C) to the warm room (34/28 °C) after one day, and after one week, on the photosynthesis (*A*_*n*_) response to increasing of CO_2_ in the intercellular airspaces (*C*_*i*_) (**A**). The effect of saplings transition from the warm room (34/28 °C) to the cold room (16/10 °C) after one day, and after one week, on the photosynthesis (*A*_*n*_) response to increasing of CO_2_ in the intercellular airspaces (*C*_*i*_) (**B**). The effect of saplings transition from the cold room (16/10 °C) to the warm room (34/28 °C), and from the warm room (34/28 °C) to the cold room (16/10 °C) after one day, and after one week, on the stomatal conductance to water vapor (*g*_*sw*_) (**C**), mesophyll conductance to CO_2_ (**D**), and calibrated electron transport rate (ETR_cal_) (**E**).

The short-term response to a change in growth temperature can be seen in Fig. 6. One day after plants were transferred from the cold room 16/10 °C to the warm room 34/28 °C, a 5% reduction in photosynthesis was observed (Fig. 6A). After 1 week, an 88% increase in photosynthesis was observed, which reflects strong fast acclimation to the warmer growth temperature conditions. The photosynthesis rate of the plants that transferred from the cold to the warm room reached 70% of the average photosynthesis rate measured in plants grown in the warm room. The values of mesophyll conductance and electron transport rate for plants that transferred from the cold to the warm room (16/10 °C–34/28 °C) were similar to those of plants that grew in the warm room (34/28 °C) (Fig. 6D,E).

When we tested the opposite process (Fig. 6B), in which plants were transferred from the warm room to the cold room (34/28 °C–16/10 °C), a 95% reduction in photosynthesis was observed after 1 day, and 98% after 1 week. The stomatal and mesophyll conductance reduced to nearly zero after 1 day. While stomatal conductance recovered after 1 week (reaching values similar to plants that grew in the cold room (16/10 °C), the mesophyll conductance remained zero (Fig. 6C,D).

### The effect of temperature on plant water relations

**Figure 7:**
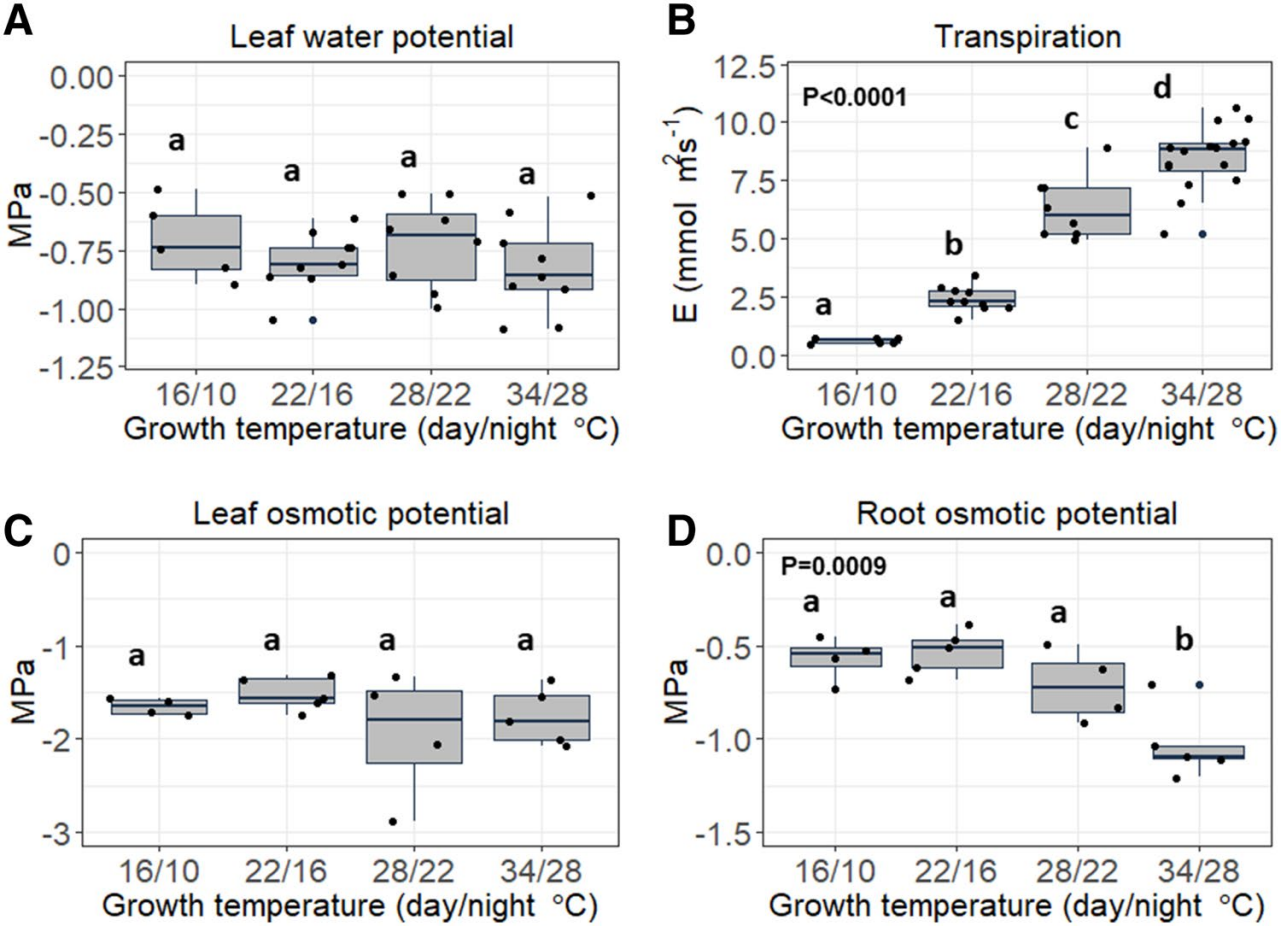
The effect of growth temperature on (**A**) the saplings leaf water potential (*Ψ*_*w*_), (**B**) transpiration, (**C**) leaf osmotic potential (*Ψ*_*s leaf*_) and (**D**) root osmotic potential (*Ψ*_*s root*_). Different letters represent a significant difference between treatments, as determined using the Tukey–Kramer post hoc test.

Growth temperature had no effect on leaf water potential (Fig. 7A). The transpiration varied significantly between the growth temperature treatments (Fig. 7B). Transpiration was highest in plants grown under high temperatures (34/28 °C, 8.4 mmol H_2_O m^−2^ s^−1^) and decreased by 25.3%, 71.4% and 92.6% in the 28/22 °C, 22/16 °C and 16/10 °C treatments respectively. Additionally, growth temperature had no effect on leaf osmotic potential (Fig. 7C). Interestingly, root osmotic potential was 30–40% lower in the warm treatment (34/28 °C) compared to other treatments (Fig. 7D).

### Seed germination in the field

**Figure 8:**
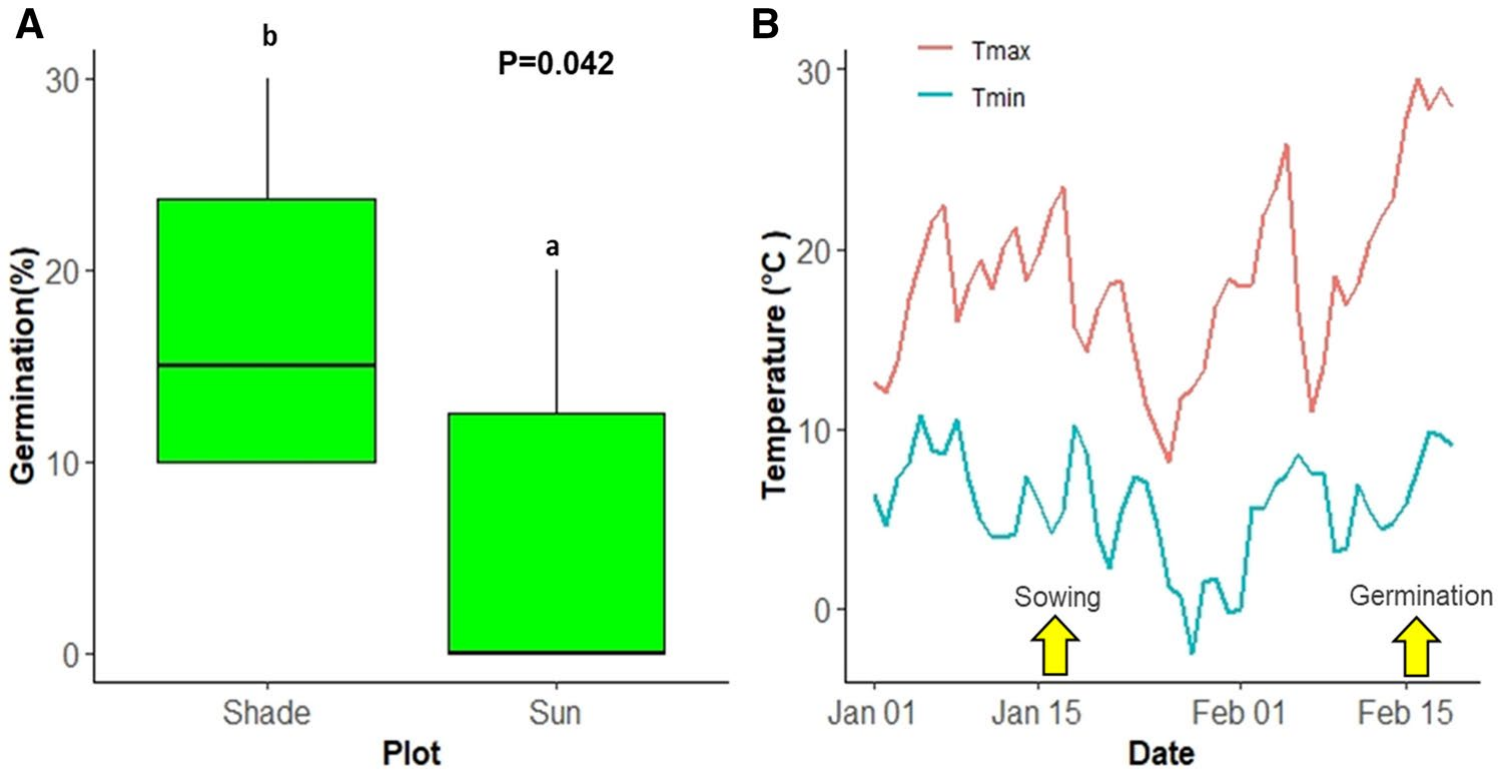
(**A**) Germination rates of seed that was sown at the field site on 18 January and which started to germinate on 16 February under the canopy of a *Z. spina-christi* tree (shade) and in an open area about 3–5 m away from the canopy (sun). Different letters represent a significant difference as determined using the Student's *t*-test (*n* = 10, *P* = 0.042). (**B**) Maximum and minimum temperatures during the field study. The arrow on the left marks the sowing date (18 January), and the arrow on the right marks the date of germination (16 February).

In the middle of January, plots of *Z. spina-christi* seeds were sown, as described above, in the open field and under the shade of a large *Z. spina-christi* tree. Germination began on 16 February, about 30 days after sowing (Fig. 8A). Germination occurred after the maximum temperature had exceeded 28 °C and the minimum temperature was above 9 °C (Fig. 8B). Important rain events occurred on 8 January (23 mm), between 19 and 26 January (62.8 mm) and between 6 and 8 February (59.8 mm). Therefore, it was presumed that the soil was fairly wet during the germination period (data not shown). The percentage of maximum germination was significantly higher in the shaded plots (17.5 ± 3.5%) compare to the sun-exposed plots (6.4 ± 3.2%).

## Discussion

The results of this study show that slow seedling growth, and not germination, is the main barrier for the successful establishment of *Z. spina-christi* under low temperature. Higher winter temperatures, as predicted for the Mediterranean in the next decades^30^ may lead to earlier germination of *Z. spina-christi*, causing seedlings to be exposed to longer periods of high soil moisture. Earlier onset of the establishment period enables seedlings to develop deeper root systems, which increases their chances of survival during the dry months of summer. Thus, the rapid germination and growth rate observed in the high-temperature treatments (34/28 °C and 28/22 °C) could help reduce mortality due to desiccation. When soil moisture is readily available, the faster the seeds germinate, and the faster the seedlings become established, the better their odds for survival. Still, seedling survival depends on many other factors, including the timing of chill and frost events during the winter, the tree’s thermal optimum for growth and nutrient availability. Our results demonstrate that early germination is not enough for successful establishment as *Z. spina christi* growth rate is inhibited by 71–77% (for both seedling and saplings) under 22/16 °C and cannot perform growth under the temperature of 16 °C (Figs. 1, 3). Thus, seedling survival depends on the correct balance between the minimum average temperatures and the number of chill events (0–10 °C) in the winter, and water availability in the spring and summer. Under natural conditions in the eastern Mediterranean region, *Z. spina-christi* typically propagates during late winter, when soil moisture is still high, and the temperature is above a certain threshold. According to our observation, that threshold was found to be 28 °C (Fig. 8). However, under natural conditions, germination and seed establishment are harder to predict, since factors such as soil fauna, microorganisms, soil moisture, soil salinity, light availability and temperature fluctuations can all cause substantial variations.

The slow growth rates for both seedlings and saplings (Figs. 1, 3) can be explained by photosynthesis limitation (Figs. 5, 6, 7). Under cold stress conditions, photosynthesis rate decreases since the low temperature affect both carbon fixation and electron transport in the thylakoids (Fig. 5). The optimal temperature for photosynthesis in young saplings found to be 34 °C, while photosynthesis is completely inhibited in temperatures of less than 16° C. The window of optimal temperatures is likely to be even narrower for seedlings than for mature trees since seedlings are typically shorter, have less leaf area, less palisade mesophyll and lower nitrogen contents. Thus, adaptation and acclimation to low temperatures may result from a tradeoff between growth optimum and the potential to tolerate cold^31^. Species can tolerate low temperatures including the increase of photosynthetic enzyme contents and preservation activity of cell membranes^32^. In this case, it appears that the genetic tropical climate background of *Z. spina-christi* does not cover a wide enough range to tolerate low temperatures. The fact that plants that transferred from warm (34/28 °C) to cold temperature (16/10 °C) were not recovered after 1 week (Fig. 6B), suggest that *Z. spina christi* does not possess mechanisms to protect its photosynthetic machinery from chill damage. As may be seen from the photosynthesis response curves (A_n_/C_i_ and the A_n_/C_c_), the mesophyll conductance (*g*_*m*_) is the main limiting factor for photosynthesis in *Z. spina-christi* under low temperatures while the Rubisco limitation (i.e. biochemical limitation) plays a minor role (Fig. 5A,B). Here we further showed that under low temperature the *g*_*m*_ is hyposensitive to an increase of CO_2_ (Fig. 5C) Also, the fact that *g*_*m*_ returned to its highest value one day after plants were transferred from a cold room to a warm one (Fig. 6C), suggests that *g*_*m*_*-*temperature dependency *in Z. spina-christi* involves metabolic regulation. Still, the underlying mechanisms that mediate reduction of *g*_*m*_ under low temperatures in thermophilic plants require further investigation.

An additional limiting factor that may explain the slow growth of the sapling under low temperatures arising from cambial dormancy, as temperature is a key factor determining the seasonality of cambium activity and secondary growth. In this study, we were able to show the strong effects of low temperatures on the anatomical properties of the vascular tissues of young saplings (Table 2). This suggests substantial temperature limitation on cambial activity of *Z. spina christi*. One interesting characteristic of the *Z. spina christi* xylem structure is that it lacks clear patterns of cambial activity and annual rings. Liphschitz and Waisel^33^ showed that in places where the level of soil moisture is relatively high even during the summer, two annual cycles of cambial activity, flowering, and seed production are observed, but during winter and spring cambium is not active. This claim is in line with a recent study showing that cambial growth of *Acacia raddiana* and *Acacia tortilis* in hyper-arid areas is completely dormant during the cold (and wet) season, and active during the summer dry season when temperatures reach a daily maximum of 45 °C^10^.

Low temperatures were also associated with an increase of the root to shoot ratios (Fig. 3), indicating that low-temperature limits shoot growth more than root growth. Increased root to shoot ratios may be an important factor in the ability of a plant to overcome water limitation under low temperatures. In our case, there was no discernible difference in the results obtained by different treatments. In all treatments. leaves were fully turgid, and the leaf water and osmotic potentials were statistically similar (Fig. 7), indicating no severe water limitation under low temperature. It is important to note that *Z. spina christi*, like other African savanna trees, is known to be drought resistant, mostly due to its ability to maintain sufficient water supply during the dry seasons as a result of a deep root system submerged in deep water sources^17^.

Germination of *Z. spina-christi* seeds depends on both temperature and physical dormancy. The physical dormancy imposed by the hard, woody endocarp was found to inhibit the rate of germination by about 50% (Fig. 2). The physical dormancy imposed by the impermeable “stony” endocarp of *Z. spina-christi* was also described in earlier reports^20–22^. In general, in these previous studies, all attempted mechanical and acid scarification methods improved the rate of emergence, but there is no clear conclusion as to which method is most beneficial for seedling establishment. Saied et al.^34^ showed that seeds that had been immersed in 97% sulfuric acid for 120 min had a 50% higher germination capacity than untreated seeds. In our case, acid scarification was found to be less effective and improved the germination rate by merely 10%. There is existing evidence that germination of *Z. spina-christi* seeds extracted from the stomachs of cattle and pigs or collected from animal droppings germinate better than control seeds (50–60% germination as compared to 30% germination^23^). Also, goats that eat the fruit tend to regurgitate the seeds, supporting both seed dispersal and germination. The fact that the hard *Ziziphus* endocarp may prevent seed germination under unfavourable conditions hints to this plant’s native Sudanese habitat, where the tree can withstand an 8–10-month-long dry season. In a study that was conducted with *Z. lotus* using PEG-6000 solutions as osmolytes, under mild drought stress (when osmotic potential of the soil solution was between 0.2–0.6 MPa), 95% of the seeds germinated. In addition, germination was severely inhibited (< 10% germination) when the osmotic potential of the soil solution was between − 0.8 and − 1 MPa^22^. However, to our knowledge, no data are currently available on the effect of soil drought conditions on the germination of *Z. spina-christi* seeds. As it is expected that in future decades, the volume and distribution of winter rains may become less predictable, further research on how *Z. spina-christi* seedlings become established under water-limited conditions is needed.

In Addition, limiting factors affecting growth and establishment depends on local conditions and different environmental factors that may interact with one another^35^. Therefore, it is hard to predict how far climate change would push the distribution limits of this species. On a global scale, we expect that if climate change trends will continue, an increase of 2–3 °C in average winter temperatures may be enough to cause substantial shifts in altitude and latitude along the Syrian–African transform, and in other tropical and subtropical regions of the world. Yet, comprehensive large-scale biogeographical studies are needed for a better understanding how far it could spread geographically in the future. It is important to note that *Z. spina-chrisi *propagates by seeds and therefore owns a wide genetic variability which may allow rapid adaptation to a changing environment^17^. Since in the current study we only focus on one ecotype, it would be interesting to study how different *Z. spina-christi* ecotypes adapt to new environments.

## Conclusions

**Figure 9:**
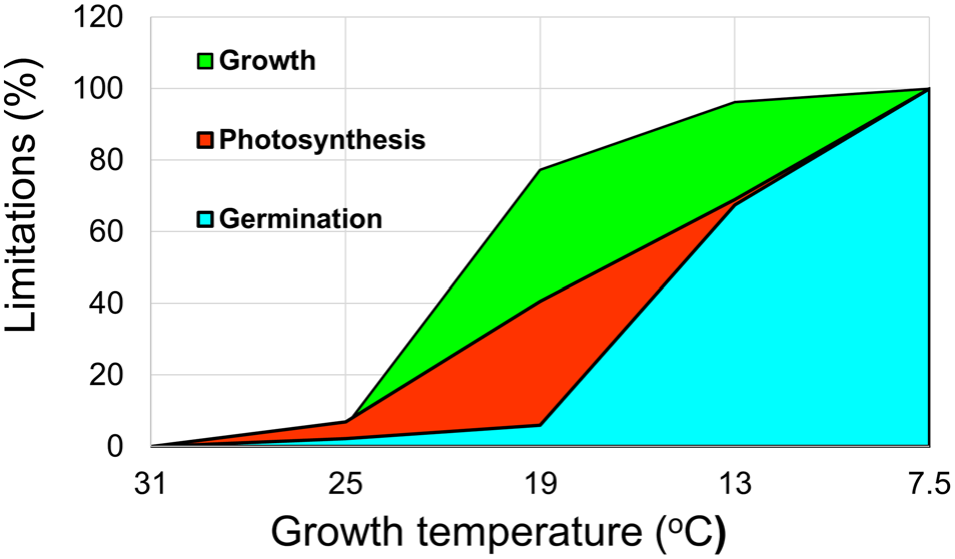
Effects of different growth temperatures on the overall limitation for successful establishment resulting from germination, photosynthesis, and growth. The temperature calculated from the average of the day/night temperature of each treatment.

This study defines the optimum thermal ranges for germination, growth, and photosynthesis, and demonstrates how low temperatures negatively affect seedling survival of *Z. spina-christi*. Growth and photosynthesis are the main limiting factors for successful establishment since their biochemical and physiological processes are more prone to cold stress than germination (Fig. 9). Higher average winter temperatures, and reduction in chill or frost events due to climate change, may give ecological advantage to *Z. spina christi* and other thermophilic trees. This may lead to earlier establishment of the seedlings during the winter and increase their chances of survival in the following summer. If our hypothesis is correct, we would expect that, in coming decades, *Z. spina-christi* may spread to higher altitudes and latitudes across the Mediterranean region.
